# An easy method of attachment to an impacted canine

**DOI:** 10.1186/2196-1042-14-11

**Published:** 2013-06-03

**Authors:** Rekha Mittal, Deepak Rai, Anand Patil, Ashish Garg

**Affiliations:** Department of Orthodontics, Manav Rachna Dental College, Faridabad, Haryana 121006 India; 210, Ashoka Enclave Main, Sector 35, Faridabad, Haryana India; Department of Orthodontics, SDM Dental College, Dharwad, Karnataka India; Department of Maxillofacial Surgery, Manav Rachna Dental College, Faridabad, Haryana 121006 India

## Abstract

**Background:**

Since many years, various bonding attachments have been used as a mode of traction for surgically exposed impacted teeth. It has always been a challenge to select an attachment considering predictability of the bonded attachment, mucogingival and periodontal conditions of the overlying tissues, and additional inventory requirement.

**Methods:**

A 0.010-in. stainless steel ligature wire with eyelets at one end and spiral twisted at the other end was made and used as an attachment to guide surgically exposed impacted canine.

**Results:**

Orthodontic guided eruption of an impacted canine of a 16-year-old patient using this simple attachment with 1-year follow-up illustrates adequate amount of attached gingiva.

**Conclusions:**

Ligature wire attachment is a simple inexpensive attachment that can be custom made without any need for additional inventory, besides being more comfortable to patients.

## Background

Over many years of orthodontic history, various methods of attachment have been used for bonding impacted tooth during closed eruption technique [1]. However, various problems like failure of bonding or tearing of mucosa due to prominent attachment have remained consistent [2].

A simple and inexpensive attachment technique has been developed to reduce the bond failure rates and discomfort to the patient during orthodontic tooth movement of an impacted teeth. Low profile of the attachment and its excellent adaptability to tooth surface is advantageous specially during tunnel traction technique [3, 4] wherein impacted teeth has to pass between two normally placed cortical plates towards the center of the alveolar ridge [5].

## Methods

**Figure 1:**
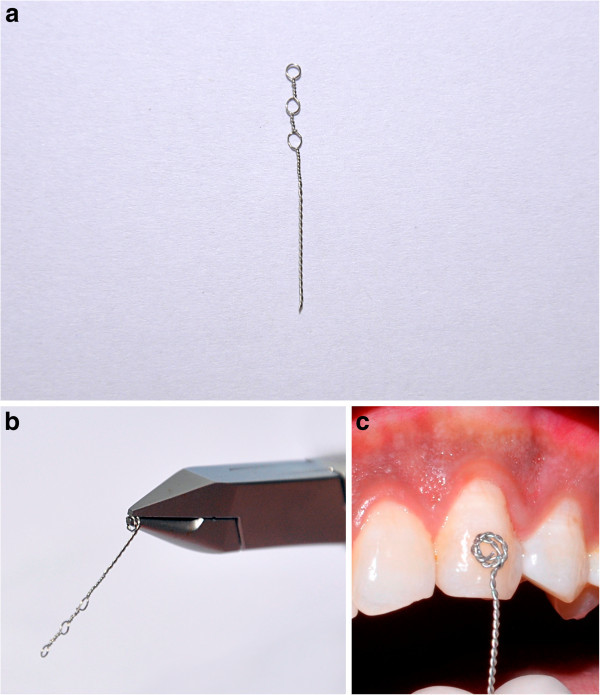
**Stainless steel ligature wire.** (**a**) A 0.010-in. ligature wire with three eyelets. (**b**) Ligature wire with spiral at one end and eyelets at the other end. (**c**) Illustrating close adaptation of spiral to tooth surface.

A 0.010-in. stainless steel (SS) ligature wire was used to make an attachment for guiding the impacted canine into the occlusion. Three eyelets (Figure 1a) are made with the 0.010-in. SS ligature wire followed by twisting of the same wire into a spiral at the end using a bird beak plier (Figure 1b). The free end of the wire with spiral was closely adapted on the exposed tooth surface with the help of Howe's plier (Figure 1c). The other end with eyelets can be used to pass an elastic thread that can be tied to the main arch wire. This attachment was placed over the etched enamel, and a flowable composite was used to bond it in place. Strength of the bonding can be checked by pulling the ligature with a firm pressure. Bond strength is adequate as the continuous spiral provides large coverage area and meshwork for attachment.

## Results and discussion

**Figure 2:**
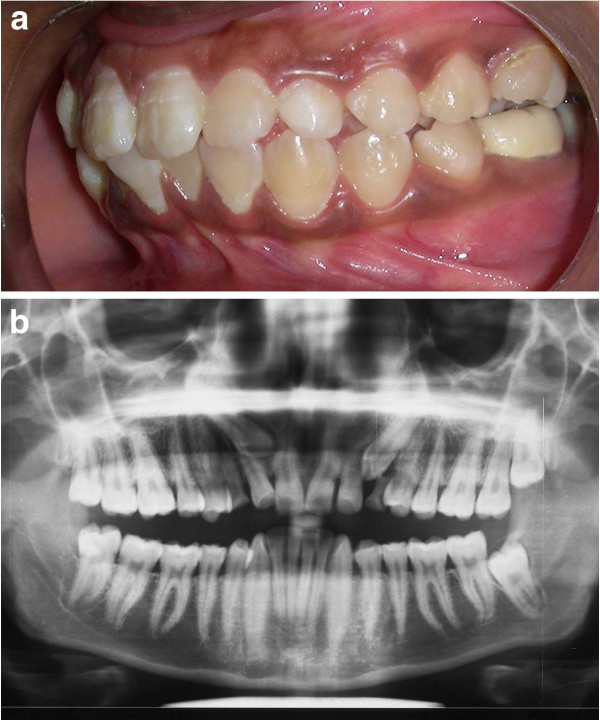
**Intraoral photograph and radiograph.** (**a**) Pretreatment intraoral photograph depicting retained deciduous canine. (**b**) Panoramic radiograph revealing impacted canine.

A 16-year-old female patient presented to us for treatment of crowding in the lower arch. A retained deciduous canine was found in the upper left anterior region (Figure 2a). The panoramic radiograph (Figure 2b) revealed impacted canine which was placed buccally.

**Figure 3:**
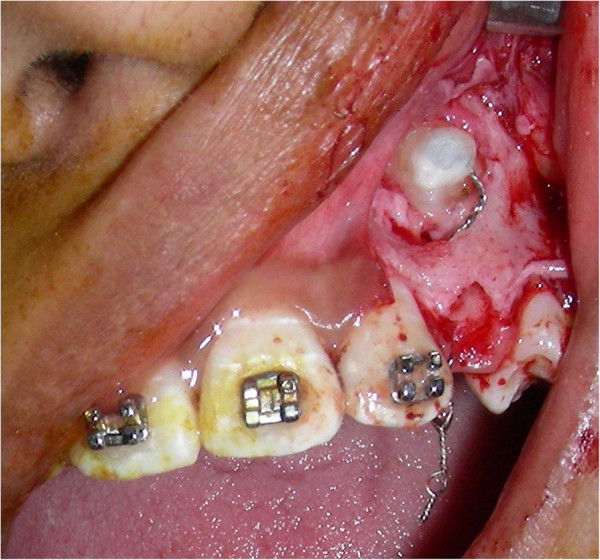
**During tunnel traction technique attachment placed over exposed canine with one end projecting into the oral cavity.**

The retained deciduous canine was extracted. Tunnel traction technique was used so that the eruption will simulate a physiologic eruption. The incisal two third of the crown was exposed by cutting a window on the cortical bone. A simple and economic method of attachment as illustrated in Figure 3 was used. One end of the attachment having spiral was placed over the tip of the exposed canine, and the other end with eyelets was passed through the tunnel and socket to emerge into oral cavity (Figure 3).

**Figure 4:**
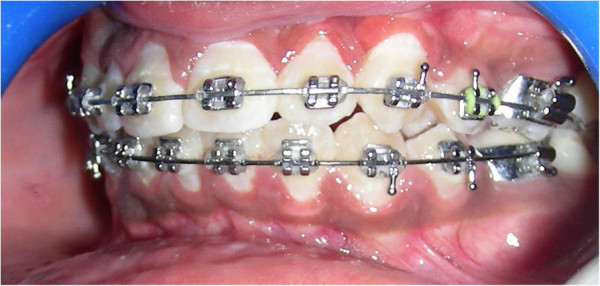
**Canine in arch during fixed mechanotherapy.**

**Figure 5:**
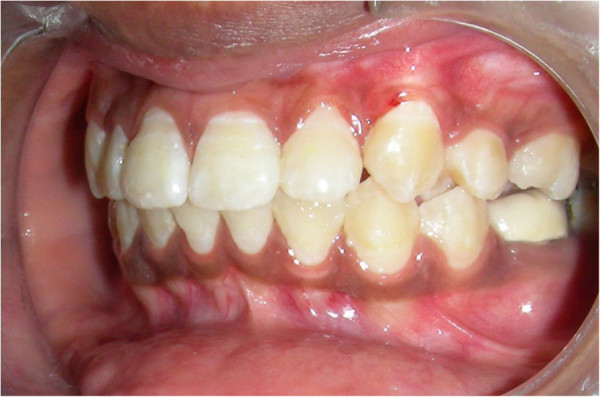
**Immediately after debonding with sufficient amount of attached gingiva.**

**Figure 6:**
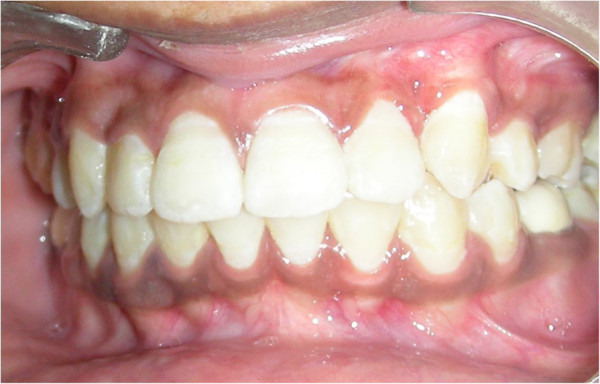
**One-year follow-up of canine showing adequate amount of attached gingiva.**

Within 6 months, guided canine eruption was achieved and had sufficient attached gingiva (Figures 4 and 5). One-year follow-up showed adequate attached gingiva with no recession (Figure 6).

## Conclusion

Ligature wire attachment is extremely easy to adapt to any impacted tooth with variable anatomy to provide a wide contact area for successful bonding. Since it is custom made with minimum projection outside the contour of crown, it is highly acceptable and comfortable for the patient.

## Consent

Written informed consent was obtained from the patient for publication of this report and accompanying images.
